# Retrograde stapling of a free cervical jejunal interposition graft: a technical innovation and case report

**DOI:** 10.1186/1471-2482-14-78

**Published:** 2014-10-15

**Authors:** Christina Hackl, Felix C Popp, Katharina Ehehalt, Lena-Marie Dendl, Volker Benseler, Philipp Renner, Martin Loss, Jurgen Dolderer, Lukas Prantl, Thomas Kühnel, Hans J Schlitt, Marc H Dahlke

**Affiliations:** 1 Department of Surgery, University Medical Center Regensburg, Regensburg 93042, Germany; 2 Department of Anaesthesia, University Medical Center Regensburg, Regensburg, Germany; 3 Department of Radiology, University Medical Center Regensburg, Regensburg, Germany; 4 Department of Trauma, Plastic and Hand Surgery, University Medical Center Regensburg, Regensburg, Germany; 5 Department of Otorhinolaryngology, University Medical Center Regensburg, Regensburg, Germany

**Keywords:** Gastric pull-up, Esophageal cancer, Conduit, Esophageal reconstruction

## Abstract

**Background:**

Free jejunal interposition is a useful technique for reconstruction of the cervical esophagus. However, the distal anastomosis between the graft and the remaining thoracic esophagus or a gastric conduit can be technically challenging when located very low in the thoracic aperture. We here describe a modified technique for retrograde stapling of a jejunal graft to a failed gastric conduit using a circular stapler on a delivery system.

**Case presentation:**

A 56 year-old patient had been referred for esophageal squamous cell carcinoma at 20 cm from the incisors. On day 8 after thoracoabdominal esophagectomy with gastric pull-up, an anastomotic leakage was diagnosed. A proximal-release stent was successfully placed by gastroscopy and the patient was discharged. Two weeks later, an esophagotracheal fistula occurred proximal to the esophageal stent. Cervical esophagostomy was performed with cranial closure of the gastric conduit, which was left in situ within the right hemithorax. Three months later, reconstruction was performed using a free jejunal interposition. The anvil of a circular stapler (Orvil®, Covidien) was placed transabdominally through an endoscopic rendez-vous procedure into the gastric conduit. A free jejunal graft was retrogradely stapled to the proximal end of the conduit. Microvascular anastomoses were performed subsequently. The proximal anastomosis of the conduit was completed manually after reperfusion.

**Conclusions:**

This modified technique allows stapling of a jejunal interposition graft located deep in the thoracic aperture and is therefore a useful method that may help to avoid reconstruction by colonic pull-up and thoracotomy.

## Background

Indications for cervical esophageal resection and short-distance reconstruction include limited cervical esophageal cancer, hypopharyngeal cancer invading the cervival esophagus and traumatic injury or dysfunction caused by congenital disorders, corrosive inury, or radiation damage [1,2]. Furthermore, reconstruction may be indicated as salvage surgery for failed gastric or colonic interposition grafts after prior esophagectomy when the remnant of the conduit is in good condition. While gastric pull-up and colonic interposition are standard reconstruction methods after extended esophagectomy, these techniques are invasive and less suitable for localized high cervical or hypopharyngeal reconstructions [1]. A cervical esophageal interposition graft, if technically feasible, implies lower perioperative mortality and morbidity, such as fistula or anastomotic leakage, and leads to fast postoperative recovery of functional GI continuity without reduction of quality of life by reflux, dysphagy or choking [1,2].

### History of jejunal interposition grafts

In 1907, Carrel first described the technique of an autologous free jejunal graft transplanted into the neck of dogs with microvascular anastomosis to the common carotid artery and internal jugular vein [3]. In the same year, the first successful use of jejunum for esophageal reconstruction in a human patient was described by Roux, using a pedicled jejunal graft [4]. In a review by Ochsner and Owens, losses of jejunal grafts using this technique were seen in 22%, mortality being as high as 46%, mainly as a result of inadequate blood supply to the jejunal flap [5]. Due to limited vascular length, vascularization was preserved in only 16 of 80 cases reported by Yudin in 1944 [6]. Inspired by this challenge, Longmire was the first to describe a modified technique adding microvascular anastomoses between the mesenteric vessels of the pedicled jejunal flap and the internal thoracic vessels [7]. After further refinement of microvascular surgery, Seidenberg was the first to describe the technique of a free jejunal flap [8], which at the same time was the first free flap described in humans. After further refinements, the method of free jejunal interposition shows an overall success rate of 91% today with flap survival in up to 97% of cases, an acceptable overall mortality of <5%, low prevalence of persisting leakage, fistulae or stricture (all <12%) and fast recovery of functional oral alimentation (60-90% within 16 days after surgery) [9-12].

### Technical description of jejunal interposition grafts

For free cervical jejunal interposition of limited esophageal cancer, a unilateral or bilateral incision medial to the sternocleidomastoid muscle is performed and the platysma is transsected upwards. After retraction of the sternocleidomastoid muscle and division of the omohyoid muscle, lymphadenectomy around the internal jugular vein, the common carotid artery and the vagal nerve can be performed if necessary. The cervical esophagus is dissected posteriorly and mobilized from the hypopharynx down to the upper thoracic aperture. Retaining sutures can be placed and resection is completed. After confirmation of tumor-free resection margins by frozen section, a laparotomy is performed and a jejunal loop with appropriately long vessels is identified by diaphanoscopy. After graft excision, the artery is flushed with heparinized saline. An end-to-end jejuno-jejunostomy is then performed to restore GI continuity in the abdomen. The graft is transferred into the cervical site in isoperistaltic direction and venous and arterial anastomoses are completed using the internal jugular vein and the superior thyroid artery, the thyrocervical trunk or the common carotid trunk [2]. Due to the high metabolic rate of the jejunum and therefore limited ischemic tolerance, reperfusion must be achieved as fast as possible [1]. After reperfusion, the lower and upper esophago-jejunal anastomoses are performed end-to-end or end-to-side by hand suture or stapler [13].

### Brief history of circular staplers and introduction of the OrVil® stapler (Covidien)

A first stapler-prototype, weighing 3.6 kg and needing approximately 2 hours of assembly time, was introduced in 1908 [14,15]. Production of commercially available staplers for intestinal and vascular anastomoses did not start before the 1950s [15]. The OrVil® stapling device (Covidien, USA) can be applied to create end-to-end, end-to-side or side-to-side anastomoses in both open and laparoscopic surgery. After assembly and staple formation, the stapler knife blade resects the excess tissue, creating a circular anastomosis of 21 mm or 25 mm in diameter. The anvil of the device is mounted on a 90 cm long PVC tube, thus enabling delivery through the esophagus and by endoscopic rendezvous procedures [16].

We here describe the case of a patient with a failed gastric conduit after leakage of the cervical anastomosis and development of a tracheal fistula. Three months after discontinuity resection of the anastomosis and formation of a cervical esophagostomy, reconstruction of the continuity with a free jejunal interposition graft was planned. This was difficult due to the location of the remaining gastric conduit deep in the thoracic aperture. Feasibility of a retrograde stapling procedure after endoscopic rendez-vous is described.

## Case presentation

**Figure 1:**
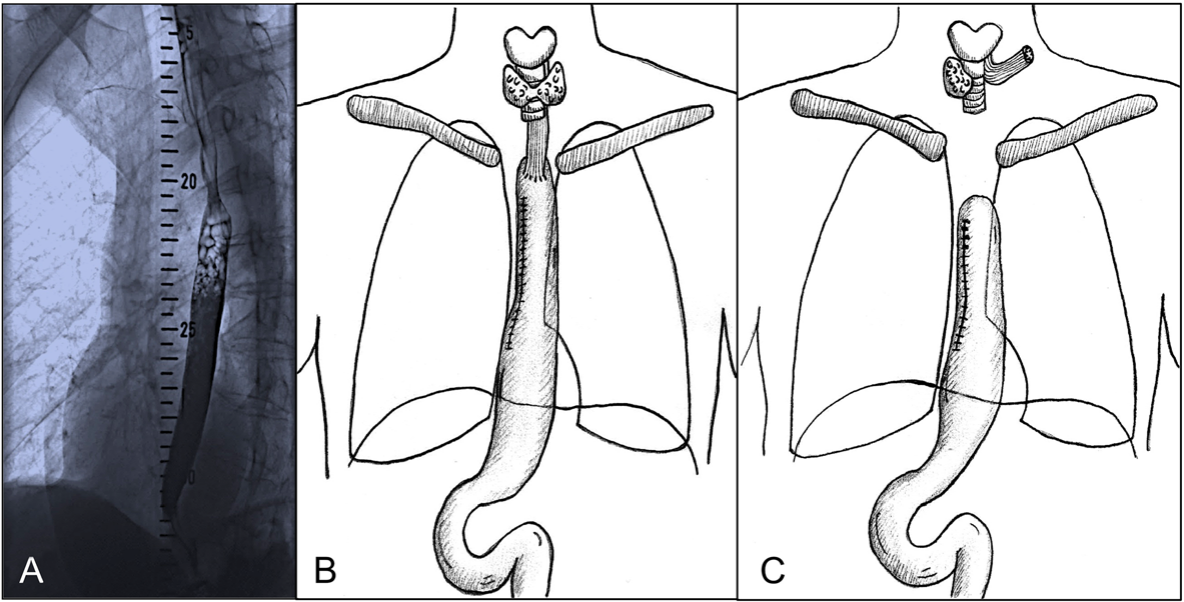
**Diagnosis of ESSC, Esophagectomy and Esophagostomy after conduit failure. A)** Workup including esophageal fluoroscopy at first presentation of the patient revealed an ESSC at 20-25 cm from the incisors. **B)** Status post Ivor-Lewis thoraco-abdominal esophagectomy with gastric pull-up and circular end-to-side stapled cervical anastomosis. **C)** Status post cervical esophagostomy and stump closure of the gastric pull-up conduit, which remained in situ within the right hemithorax.

A 56 year-old caucasian female patient was referred with the diagnosis of esophageal squamous cell carcinoma (ESSC). The patient had a history of smoking (55 pack-years) and moderate alcohol consumption. Work-up including fluoroscopy, thoraco-abdominal CT scan, gastroscopy, bronchoscopy and blood works confirmed the diagnosis of a circular ESSC at 20-25 cm from the incisors (Figure 1A) with no suspect lymph nodes and no distant metastasis. After case discussion in an interdisciplinary disease management board, the patient underwent 2 cycles of neoadjuvant radiochemotherapy (cisplatin 60 mg/m2; 5-FU 1000 mg/m2; 45 Gy). A re-staging thoracoabdominal CT scan showed significant decrease in tumor size. Ivor-Lewis thoracoabdominal esophagectomy with gastric pull-up and circular end-to-side stapled cervical anastomosis (21 mm) was then performed as planned (Figure 1B). The operation included three-field lymphadenectomy, resection of the Azygos vein, cholecystectomy and insertion of a fine-needle catheter jejunostomy (FCJ) for early postoperative enteral nutrition. Histologic analysis confirmed a ypT2, ypN0, L0, V0, R0 G2 ESCC. The patient was transferred from surgical ICU to the normal surgical ward on post-operative day 5, eating strained food, supplemented by FCJ-feeding. Due to increasing CRP and leucocytosis on post-operative day 8, a thoraco-abdominal CT scan and EGD transit were performed and an anastomotic leakage with viable perfusion of the conduit was diagnosed. A proximal-release stent was successfully placed by endoscopy. After prolonged recovery from the resulting sepsis, the patient could be discharged in good clinical condition and eating regular diet on post-operative day 56. Two weeks after discharge, the patient presented with symptoms of pneumonia and was readmitted. Workup including blood-works, thoraco-abdominal CT scan, bronchoscopy and gastroscopy revealed an esophagotracheal fistula cranial to the esophageal stent, not associated with the primary cervical anastomosis. After unsuccessful tracheal stenting and no possible further interventional improvement, the fistula was resected and cervical esophagostomy was performed with tracheal reconstruction by a sternoflap. The stump of the gastric pull-up conduit was closed using 3/0 monocryl hand-sewn interrupted suture and the intrathoracic part of the conduit remained in situ within the right hemithorax (Figure 1C). The patient could be transferred from surgical ICU to the normal ward on post-operative day 4 and was discharged on post-operative day 27. At routine follow-up three months later, the patient presented in good general condition. Restaging remained without new evidence of disease and free jejunal interposition was scheduled.

### Operative Technique modifying the Free Jejunal Interposition

**Figure 2:**
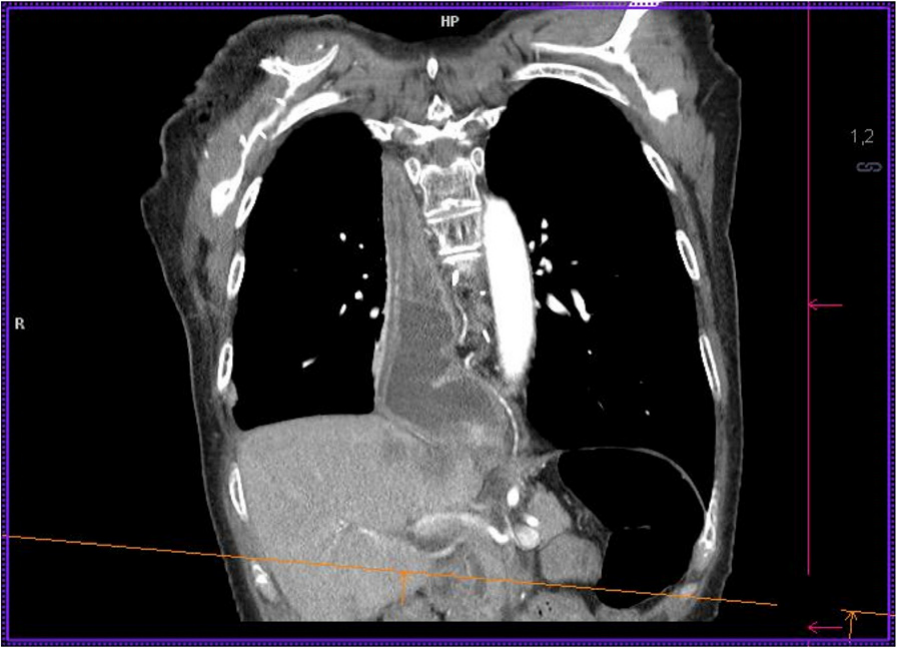
Position of the gastric pull-up conduit retracted into the right hemithorax before reconstruction.

**Figure 3:**
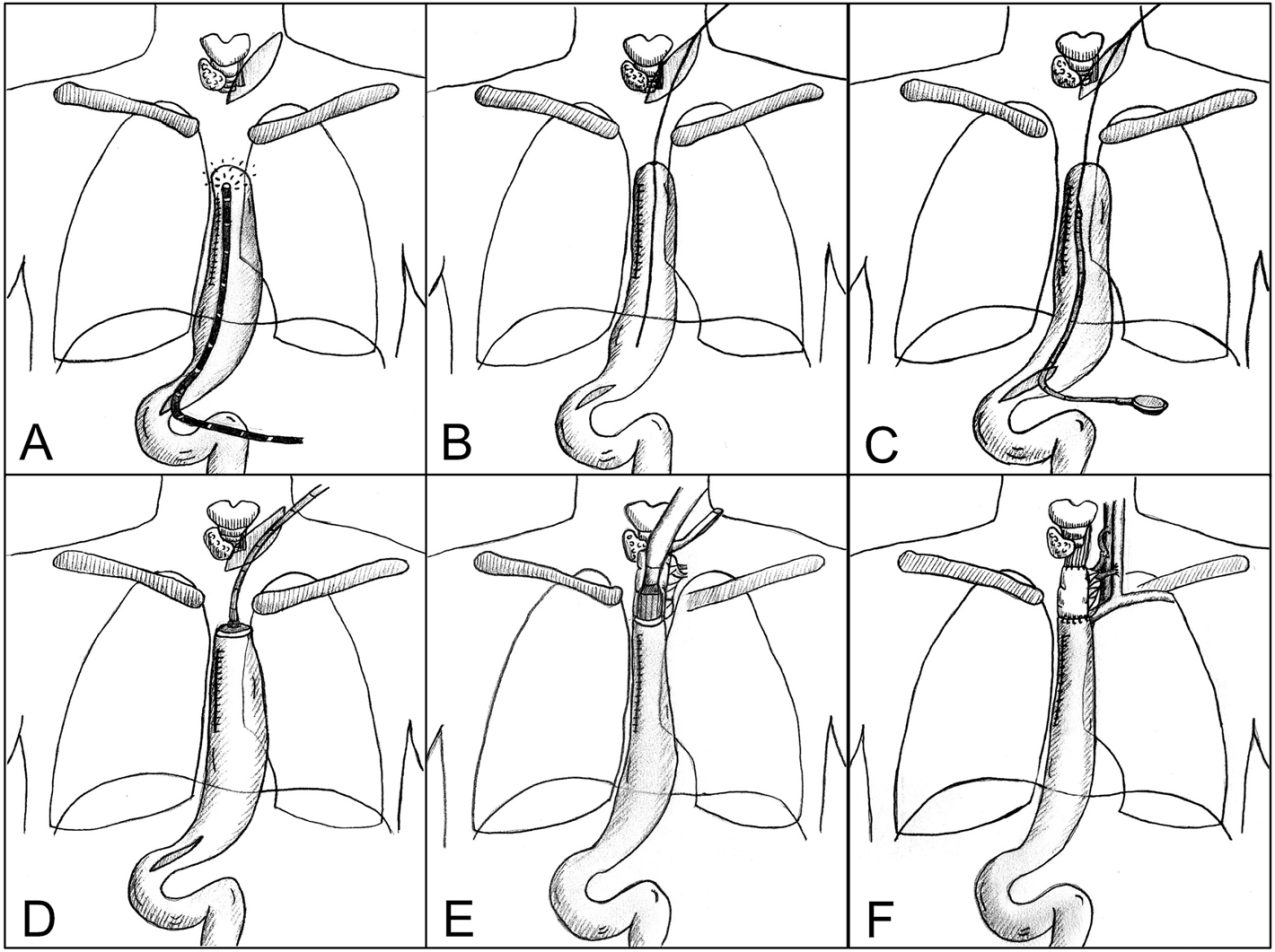
**Reconstruction with free jejunal interposition. A)** Gastroscopy and diaphanoscopy of the pull-up gastric interposition after gastroscope insertion via the distal conduit. **B)** Guide-wire positioning from the cervical incision into the gastric pull-up conduit by endoscopic rendez-vous. **C)** Attachment of the OrViL® delivery tube to the guide-wire. **D)** Insertion of the OrViL® circular stapler into the pull-up gastric interposition **E)** Stapling of the jejunogastrostomy. **F)** Revascularization and proximal anastomosis of the free jejunal interposition.

**Figure 4:**
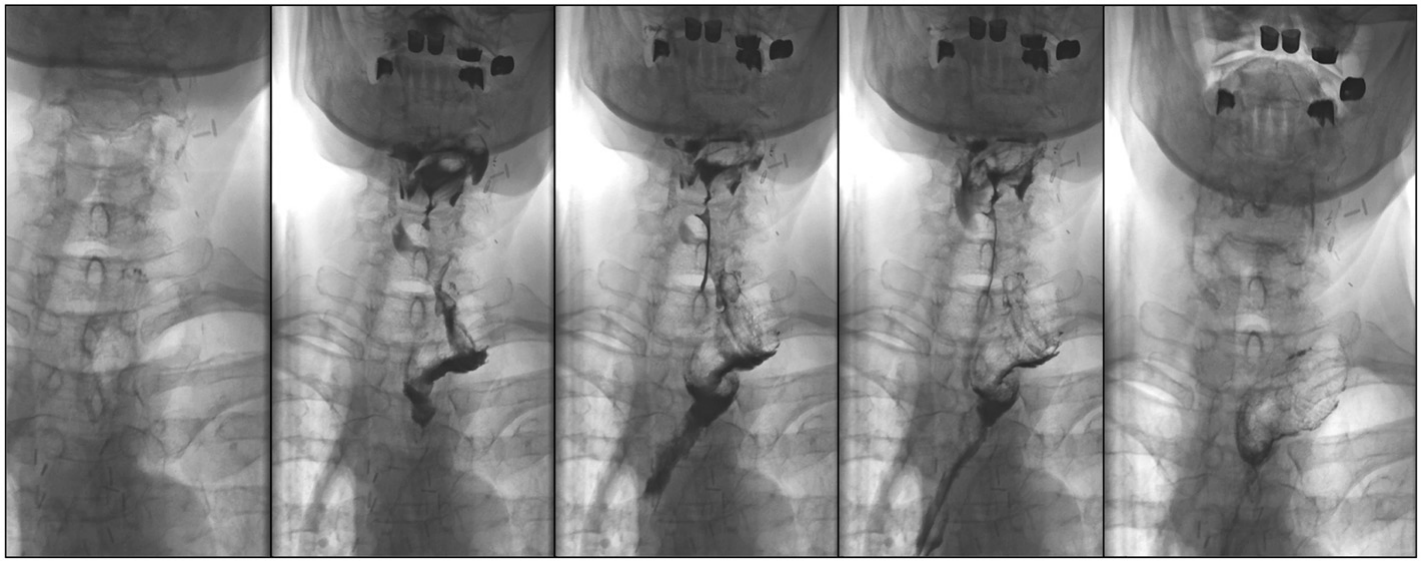
Regular EGD transit before discharge of the patient.

Angiography of SMA, IMA, ECA and subclavian arteries showed no pathological findings. Intraoperatively, the proximal stump of the gastric interposition was exposed after cervical incision. Exposition was challenging due to the very deep intrathoracic position of the conduit stump (Figure 2). Therefore, a laparotomy was performed and an endoscopy of the pull-up gastric interposition was performed after gastroscope insertion via the distal part of the gastric conduit (Figure 3A). After endoscopic diaphanoscopy, a guide-wire was placed by endoscopic rendez-vous from the cervical incision into the gastric pull-up conduit (Figure 3B) and the OrViL® delivery tube was attached to the guide-wire (Figure 3C). Then, a suitable jejunal loop with vascular pedicle was identified, excised, flushed with ice-cold heparine/saline, kept on ice and transferred to the cervical operation site. The OrViL® circular stapler was inserted into the aboral part of the free jejunal graft (Figure 3D,E) and stapling of the jejunogastrostomy was performed (Figure 3E). Venous and arterial anastomoses were then performed by side-to end venous anastomosis using the internal jugular vein and end-to end arterial anastomosis using the left superior thyroid artery. Total ischemic time of the graft was less than 60 minutes with continuous cooling. After reperfusion, the jejunal graft appeared viable pink with increased peristalsis. The small incision at the distal gastric conduit used to insert the gastroscope was closed with 3/0 monocryl hand-sewn interrupted suture. Pharyngojejunal end-to-side anastomosis was then performed by hand with interrupted sutures after reperfusion (Figure 3F). Three centimeters of the cervical incision were left open to monitor the graft. The patient could be transferred from surgical ICU to the surgical ward on post-operative day 8. On day thirteen, a secondary wound closure was undertaken, which resulted in minimal leakage of the proximal anastomosis. The patient was discharged home on post-operative day 44 with regular EGD transit (Figure 4). Two weeks after discharge, the patient presented with stricture of the jejunal interposition at the distal anastomosis, which could successfully be resolved by single endoscopic balloon dilation therapy. Another three weeks later, the cervical incision had completely healed and the patient tolerated small portions of regular diet with slow weight gains. One year after final discharge, the patient presented tumor-free and eating regular diet.

## Conclusions

We here describe a modified technique for retrograde stapling of a free jejunal interposition graft for a failed gastric pull-up. The gastric conduit had remained in situ within the right hemi-thorax, its proximal end deep in the thoracic aperture, opening the possibility of limited-length reconstruction by free jejunal interposition. During reconstruction, cervical exposition of the proximal gastric stump was challenging due to its position deep in the thoracic aperture. Preparation for hand-sewn distal anastomosis would have meant a traumatizing access by sternotomy and/or thoracotomy, including a significant risk of surgical damage and yet accepting a high risk of anastomotic leakage. Instead, a laparotomy was performed with subsequent distal gastrostomy and insertion of a gastroscope followed by retrograde gastroscopy of the pull-up gastric interposition. By diaphanoscopy, the proximal stump of the gastric conduit was safely identified and a guide-wire was inserted from the cervival incision site. Then, the anvil of the OrViL circular stapler was introduced via the conduit and the jejuno-gastrostomy was stapled prior to microvascular reconstruction of the graft vessels. Concerning esophagogastric surgery with jejunal interposition, an ongoing controversy exists, whether esophago-jejunal or pharyngo-jejunal anastomoses should preferably be performed as end-to-side circular stapling or be hand-sewn end-to-end [13,17]. Up until now, no significant differences in anastomotic leakage rates are seen whereas the influence on long-term anastomotic strictures is a matter of debate [17].

In our case, revascularisation was performed after the jejuno-gastrostomy, thus enabling adaptation of an ideal vessel-length with the jejunal interpostion already in situ. Vice versa, stapling of the jejunogastrostomy, being a fast and safe procedure, did not necessitate prior microvascular anastomosis of the ischemia-intolerant jejunal flap. Leaving the gastric conduit in situ thus enabled salvage surgery by interposition of a short jejunal segment, offering the benefits of excellent peristalsis, absence of alkaline or acidic reflux and excellent size match of the two conduits [1].

Alternative methods for reconstruction of the cervical esophagus after failure of a gastric conduit are pectoralis major flaps or free tissue transfers such as the radial forearm flap or the anterolateral thigh flap or colonic interposition [1,18]. The pectoralis major flap as well as free tissue transfers offer excellent length, are resistant to reflux, allow functional swallowing and show low cardiopulmonary co-morbidity. Furthermore, no abdominal surgery is needed for flap harvest. In contrast, pectoralis major or free tissue flaps do not enable peristalsis, often show long long-term redundancy and significant risk of strictures and fistulas in the longer term [1,18]. Colonic interpositions offer the benefits of excellent length and long-term function and would be ideal for bridging long defects of the thoracic esophagus. On the other hand, colonic interposition implies the risk of extended abdominal surgery, complicated blood supply, intrinsic diseases such as cancer or bleeding, and long-term redundancy with a reduced overall quality of life [1,19].

Taken together, we here describe a modified technique of retrograde stapling of a jejunal interposition graft to a gastric conduit that had remained in situ after a leaking esophageal anastomosis. This technique may be of value for a selected group of patients with this unique surgical problem and may help to avoid then unnecessary thoracotomies or colonic pull-ups.

## Consent

Written informed consent was obtained from the patient for publication of this Case report and any accompanying images. A copy of the written consent is available for review by the Editor of this journal.

## Competing interests

The authors declare that they have no competing interests.

## Authors’ contributions

CH drafted, wrote an edited the manuscript and edited the figures. FCP helped to dritically revise the manusript and performed parts of the surgeries. KE and LMD created the figures presented in this manuscript. VB and PR helped drafting and critically revising the manuscript. ML, JD, LP, TK, HJS and MHD were the surgeons performing this new technique of retrograde stapling of a jejunal interposition graft to a gastric conduit. Furthermore, MHD drafted and critically revised the manuscript and HJS critically revised the manuscript. All authors read and approved the final manuscript.

## Pre-publication history

The pre-publication history for this paper can be accessed here:

http://www.biomedcentral.com/1471-2482/14/78/prepub
